# Oct4 Gene Expression in Primary Colorectal Cancer Promotes Liver Metastasis

**DOI:** 10.1155/2019/7896524

**Published:** 2019-05-02

**Authors:** Shiki Fujino, Norikatsu Miyoshi

**Affiliations:** ^1^Innovative Oncology Research and Regenerative Medicine (iNOR), Osaka International Cancer Institute, 3-1-69, Ohtemae, Chuo-ku, Osaka 541-8567, Japan; ^2^Department of Gastroenterological Surgery, Graduate School of Medicine, Osaka University, 2-2-E2, Yamadaoka, Suita, Osaka 565-0871, Japan

## Abstract

**Purpose:**

The *Oct4* gene plays an important role in undifferentiated embryonic stem cells and regulates stem cell pluripotency. The aim of this study was to examine the relationship between Oct4 expression and liver metastasis of colorectal cancer (CRC) in clinical samples and investigate the role and abilities of Oct4-positive CRC cells.

**Methods:**

The study included 158 patients who underwent surgery for CRC between 2009 and 2011. The correlations between the *Oct4* gene expression and the clinical parameters were assessed, and liver metastasis-free survival (LMFS) was evaluated in these patients. Oct4-EGFP-positive cells were established to examine their subpopulation and ability. The capacity to form liver metastasis in vivo was examined using CRC cell lines and primary cultured CRC cells.

**Results:**

LMFS was significantly poor in the *Oct4* high-expression group compared with the low-expression group (*P* = 0.008). Multivariate analyses showed that *Oct4* expression (*P* = 0.015) and TNM stage (*P* < 0.001) were significantly correlated with LMFS. Oct4-EGFP-positive cells highly expressed stem cell-associated markers and had self-renewal and differentiation abilities. Oct4-high cells actively formed liver metastasis.

**Conclusion:**

The Oct4 expression was correlated with liver metastasis in CRC patients. Oct4 expression cells have self-renewal and differentiation abilities like those of cancer stem cells. Oct4 contributed to forming liver metastasis in CRC.

## 1. Introduction

Cancer is a leading cause of death in Japan and developed countries, and it has become a major cause of death in developing countries [1, 2]. It is estimated that the global total number of deaths by cancer will be 9.6 million in 2018, and colorectal cancer (CRC) will be the third leading cause of cancer death (10.2% of total cancer deaths) [2].

Distant metastasis causes death in patients with CRC, and liver metastasis is most commonly found in CRC patients [3, 4]. The development of systemic combination chemotherapy has improved the prognosis. However, the median overall survival (OS) for patients with metastatic CRC (mCRC) is approximately 30 months [5], and the 5-year survival rate is only 19% in stage IV patients [4]. It is necessary to determine the mechanism of distant metastasis to develop treatment to improve the prognosis. Identifying the mechanism and genes responsible for liver metastasis will help to control the morbidity of CRC patients.

The gene encoding the POU domain, class 5, transcription factor 1 (POU5F1), also known as Oct4, is expressed in embryonic stem cells (ES) and plays an important role in maintaining the pluripotency and self-renewal of ES cells [6, 7]. Oct4 is also expressed in tissue stem cells and is involved in their proliferation and differentiation [8, 9]. We previously reported that high Oct4 expression was a novel prognostic marker in CRC [10]. Oct4 was also related to malignancy and cancer stem cells (CSCs) in some cancers. In breast cancer, Oct4 expression levels were significantly associated with nonsentinel lymph node metastases [11], and in osteosarcoma, Oct4 was related to stem cell-like properties [12]. Oct4 promoted tumorigenesis of cervical cancer cells [13] and induced stem cell-like properties and epithelial-mesenchymal transition (EMT) in lung cancer [14]. Oct4 regulated EMT and its knockdown inhibited cell migration and invasion of CRC cell lines [15]. Oct4 is thought to play an important role in CSCs [10, 12] but only part of the mechanism is known. This study focused on the role of Oct4 in metastatic CRC (mCRC), the relationship between Oct4 expression and liver metastasis of CRC in clinical samples, and the role of Oct4-expressed cells in primary cultured cells. We aimed to investigate its roles in the prognosis of mCRC patients and reveal the stem cell-like properties of Oct4 in CRC.

## 2. Materials and Methods

### 2.1. Clinical Samples

One hundred seventy-three patients with CRC were registered. One hundred fifty-eight patients underwent complete resection of primary tumors (R0 resection, Cur A), and 15 patients underwent complete resection of primary and metastatic tumors (R0 resection, Cur B) at Osaka International Cancer Institute between 2009 and 2011 [4]. No patients received chemotherapy and/or radiotherapy before surgery. After receiving their informed consent, primary CRC specimens and normal colorectal mucosa were obtained from patients according to institutional ethical guidelines. The specimens were fixed, sectioned, and stained with hematoxylin and eosin and Elastica van Gieson stains as we previously reported [10]. The histological differentiation and lymphatic and venous invasion were examined. For gene expression analysis, surgically resected specimens were frozen in liquid nitrogen and kept at −80°C. All the patients underwent follow-up blood examinations to check tumor markers (serum carcinoembryonic antigen (CEA) and cancer antigen 19-9 (CA19-9)), and imaging examinations such as abdominal ultrasonography, computed tomography, and chest X-ray were performed every 3–6 months after surgery. According to the Japanese guidelines [4], stage III patients and stage IV patients with R0 resection received adjuvant postoperative chemotherapy after receiving informed consent.

The clinicopathological factors were diagnosed according to the tumor node metastasis (TNM) classification of the International Union Against Cancer (UICC) [16]. The Osaka International Cancer Institute Ethics Committee approved this study (no. 1608057113), and written informed consent was obtained from all patients.

### 2.2. RNA Preparation and Expression Analyses

An RNA Purification Kit (Qiagen, Hilden, Germany) was used to prepare total RNA. Reverse transcription was performed using a Transcriptor First-Strand cDNA Synthesis Kit (Roche Diagnostics, Tokyo, Japan). Designed primers and used Universal Probe Library platform (Roche Diagnostics) are listed in Supplementary Table S1. cDNA from NTERA-2 were studied as a positive control. Quantitative assessment was performed using real-time reverse transcription- (RT-) PCR using a Universal Probe Library platform (Roche Diagnostics) and FastStart TaqMan Probe Master (Roche Diagnostics) for cDNA amplification of target genes. The expression ratios of *Oct4* mRNA copies were calculated after normalization against the *GAPDH* mRNA expression.

### 2.3. Culture of CRC Cell Lines

The human colorectal tumor cell lines HCT116, DLD-1, and RKO, gifted by Dr. Bert Vongelstein (Johns Hopkins University, Baltimore, MD, USA), were cultured in Dulbecco's modified Eagle's medium (DMEM) supplemented with 10% fetal bovine serum (FBS; Thermo Fisher Scientific Inc., Waltham, MA, USA), 1% GlutaMAX-I (Thermo Fisher Scientific Inc.), and 1% penicillin/streptomycin/amphotericin B (Wako Pure Chemical Industries Ltd., Osaka, Japan). The cells were kept at 37°C in a humidified atmosphere containing 5% CO_2_.

### 2.4. Primary Culture of CRC Cells

CRC tissue was cut into 1 mm pieces and dissociated using 1 mg/mL collagenase (C6885; Sigma-Aldrich, St. Louis, MO, USA) in DMEM (Sigma-Aldrich) and shaken by a BioShaker BR-13FP (Taitec Co., Saitama, Japan) at 6 × g for 15 min at 37°C. The dissociated tissue was filtered through custom-made filters (Sansho Co. Ltd., Tokyo, Japan). It was centrifuged at 400 × g for 5 min at room temperature, and the collected cell pellet was resuspended in 2 mL culture medium (modified stem cell culture medium). Suspended primary culture cells (603siCC, 821siCC, and 28OsiCC) were seeded on plates coated with 0.03% Matrigel (Corning Inc., Corning, NY, USA) in DMEM/F12 (Sigma-Aldrich). The medium was changed every 2 or 3 days. After the cells had spread over more than 50% of the plate, they were passaged using Accutase (Nacalai Tesque, Kyoto, Japan) for about 5 min. The cells were collected and resuspended in the culture medium and seeded on a Matrigel-coated plate.

### 2.5. Xenograft Model

For the histological examination, a xenograft model was established. Accutase-dissociated cells (1 × 10^6^ cells) suspended in Matrigel (BD Biosciences) were transplanted subcutaneously into the dorsal flanks of 7-week-old nonobese diabetic/severe combined immunodeficiency mice (CLEA, Tokyo, Japan). The mice were sacrificed when the tumors reached a diameter of 10 mm. For the liver metastasis model, cells (1 × 10^6^ cells) suspended in 80 *μ*L Dulbecco's modified phosphate-buffered saline (D-PBS; Wako Pure Chemical Industries) were injected into the spleen, which was surgically resected 15 min later. Liver metastasis was evaluated 4 weeks later. The mice were weighed weekly, and none lost weight.

### 2.6. Immunohistochemistry

After deparaffinization and blocking, sections of CRC specimen were incubated with primary anti-Oct4 rabbit polyclonal antibody (#2570; Cell Signaling Technology Inc., Beverly, MA, USA) at a dilution of 1 : 200 overnight at 4°C. Vectastain Universal Elite (Vector Laboratories, Burlingame, CA, USA) was used to detect the signal. Diaminobenzidine was used for color modification. All sections were counterstained with hematoxylin.

### 2.7. Flow Cytometry and Single-Cell Sorting

The expression of surface proteins on cultured cells was measured with flow cytometry. Tumor cells were harvested upon incubation with Accutase (Nacalai Tesque). Cells were stained using CD133/1 (AC133) conjugated to allophycocyanin (APC; 130-090-826; Miltenyi Biotec, Auburn, CA) and CD44 conjugated to APC/Fire750 (33817; BioLegend, San Diego, CA). Relative fluorescent intensities were measured using an SH800 cell sorter (SONY, Tokyo, Japan). Single cells were sorted using an SH800 cell sorter (SONY). Data were analyzed with FlowJo 10.2 software (FlowJo LLC, Ashland, OR, USA).

### 2.8. Establishment of Oct4-EGFP Cells

PL-SIN-Oct4-EGFP, which expresses EGFP under Oct4 promoter, was a gift from James Ellis (Addgene plasmid # 21319). It was transfected into primary culture cells using Lentiviral High Titer Packaging Mix with pLVSIN (Takara Bio Inc., Otsu, Japan) according to the manufacturer's protocol. EGFP-positive cells were enriched by sorting twice using an SH800 cell sorter (SONY). *Oct4* mRNA expression was determined using quantitative RT-PCR.

### 2.9. RNA Analysis

Gene expression microarrays were analyzed for Oct4-EGFP cells. Oct4-EGFP-high cells and Oct4-EGFP-negative cells were sorted using an SH800 cell sorter (SONY), and total RNA was prepared using an RNA Purification Kit (Qiagen). A gene expression microarray (Agilent, Santa Clara, CA, USA) was also constructed (see Supplementary Materials). Gene set enrichment analysis (GSEA) was performed with GSEA 3.0 software (Broad Institute, Cambridge, Massachusetts, USA) to compare expression profiles of Oct4-EGFP-high cells with Oct4-EGFP-negative cells.

### 2.10. Statistical Analyses

The relationships between the *Oct4* expression and clinicopathological factors were analyzed with Wilcoxon's rank sum and *χ* ^2^ tests. Kaplan–Meier survival curves were plotted and compared by the generalized log-rank test. Univariate and multivariate analyses were performed to identify prognostic factors using a Cox proportional hazards regression model. The values of *in vitro* assays were analyzed using Wilcoxon's rank test. All statistical analyses were performed using the JMP software program (ver. 13.0.0; SAS Institute, Cary, NC, USA). A *P* value of <0.05 was considered statistically significant.

## 3. Results

### 3.1. Oct4 Expression in Clinical Samples and Clinicopathological Factors

**Figure 1:**
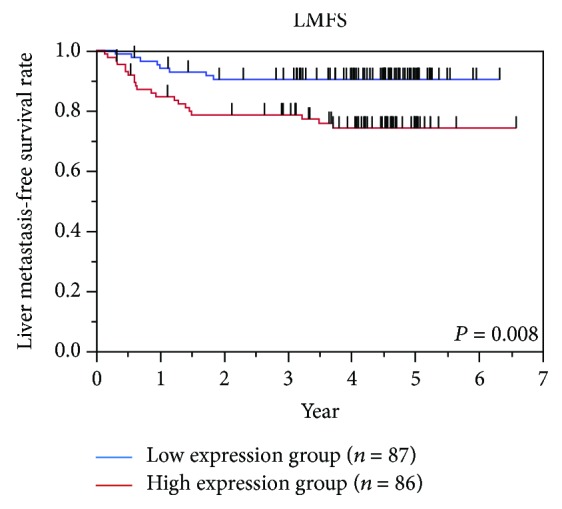
Survival curves for liver metastasis-free survival (LMFS) according to *Oct4* mRNA expression. The patients were divided into two groups according to the median value of the *Oct4/GAPDH* mRNA expression level of primary tumor. The 5-year LMFS rate was 90% (*n* = 87) in the low-expression group and 74% (*n* = 86) in the high-expression group (*P* = 0.008).

*Oct4* mRNA expression levels were determined in primary CRC using quantitative RT-PCR. *Oct4* mRNA expression levels were calculated as *Oct4*/*GAPDH* expression for each sample, and the median value of the *Oct4/GAPDH* mRNA expression level was 0.273 (range, 0.021-10.187; Supplementary Figure S1). We previously reported that *OCT4* mRNA expression levels were correlated with protein levels [10]. All patients' clinicopathological characteristics are summarized in Table 1. The patients comprised 94 males and 79 females, ranging in age from 16 to 88 years (median, 65 years). Ten patients had stage I disease, 66 patients stage II, 82 patients stage III, and 15 patients stage IV. We divided the patients into two groups according to the median value of the *Oct4/GAPDH* mRNA expression level: low expression (<0.273) and high expression (>0.273). The low-expression group included 87 patients, and the high-expression group included 86 patients. The relationships between *Oct4* expression status and clinicopathological factors are summarized in Table 2. *Oct4* expression status was not significantly correlated with any of the clinicopathological factors such as histological grade, tumor invasion, lymph node metastasis, lymphatic invasion, and vascular invasion. According to univariate analysis, high TNM stage (*P* < 0.001) and high *Oct4* expression (*P* = 0.007) were significantly correlated with poor liver metastasis-free survival (LMFS; Table 3). Multivariate regression analysis showed that high TNM stage (*P* < 0.001) and high *Oct4* expression (*P* = 0.015) were also independent predictors of poor LMFS (Table 3). Distribution of *Oct4* mRMA expression levels stratified by liver metastasis status and TNM stage is shown in Supplementary Figure S2. OS, disease-free survival (DFS), and LMFS were evaluated in all patients. *Oct4* expression was not significantly correlated with OS and DFS (Supplementary Figure S3). However, LMFS was significantly worse in the high-expression group than in the low-expression group (*P* = 0.008; Figure 1). Five-year LMFS was 90% in the low-expression group and 74% in the high-expression group.

### 3.2. Analysis of Oct4-EGFP-Positive Cells

**Figure 2:**
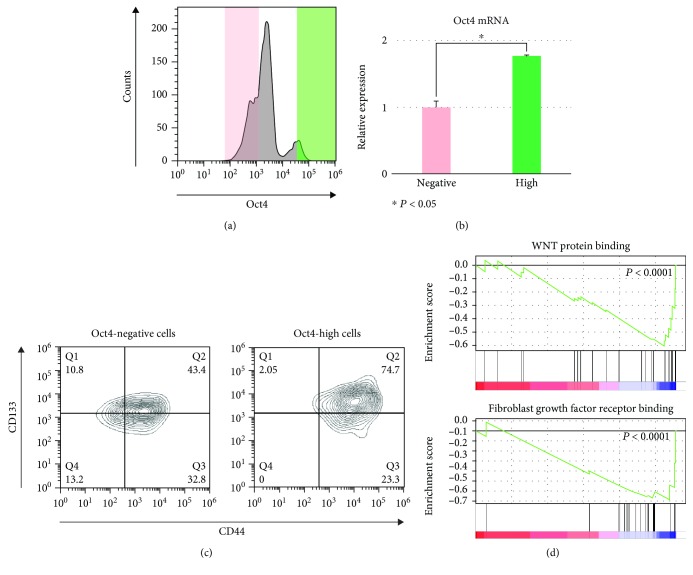
Analysis of Oct4-EGFP-positive cells. (a) Representative FACS of enriched Oct4-EGFP cells by sorting. Oct4-positive cells and Oct4-negative cells were shown. Oct4-high cells (green area) and Oct4-negative cells (pink area) were analyzed. (b) *Oct4* mRNA expression of Oct4-high cells (green area of (a)) was high compared with that of Oct4-negative cells (pink area of (a); *n* = 3, *P* < 0.05). (c) Expressions of CD44 and CD133 were high in Oct4-high cells (green area of (a)) compared with Oct4-negative cells (pink area of (a)). (d) Gene set enrichment analysis (GSEA) of Oct4-high cells (green area of (a)) and Oct4-negative cells (pink area of (a)). Representative GSEA was shown, and genes relating to WNT protein binding (*P* < 0.001) and fibroblast growth factor receptor binding (*P* < 0.001) were enriched in Oct4-high cells.

**Figure 3:**
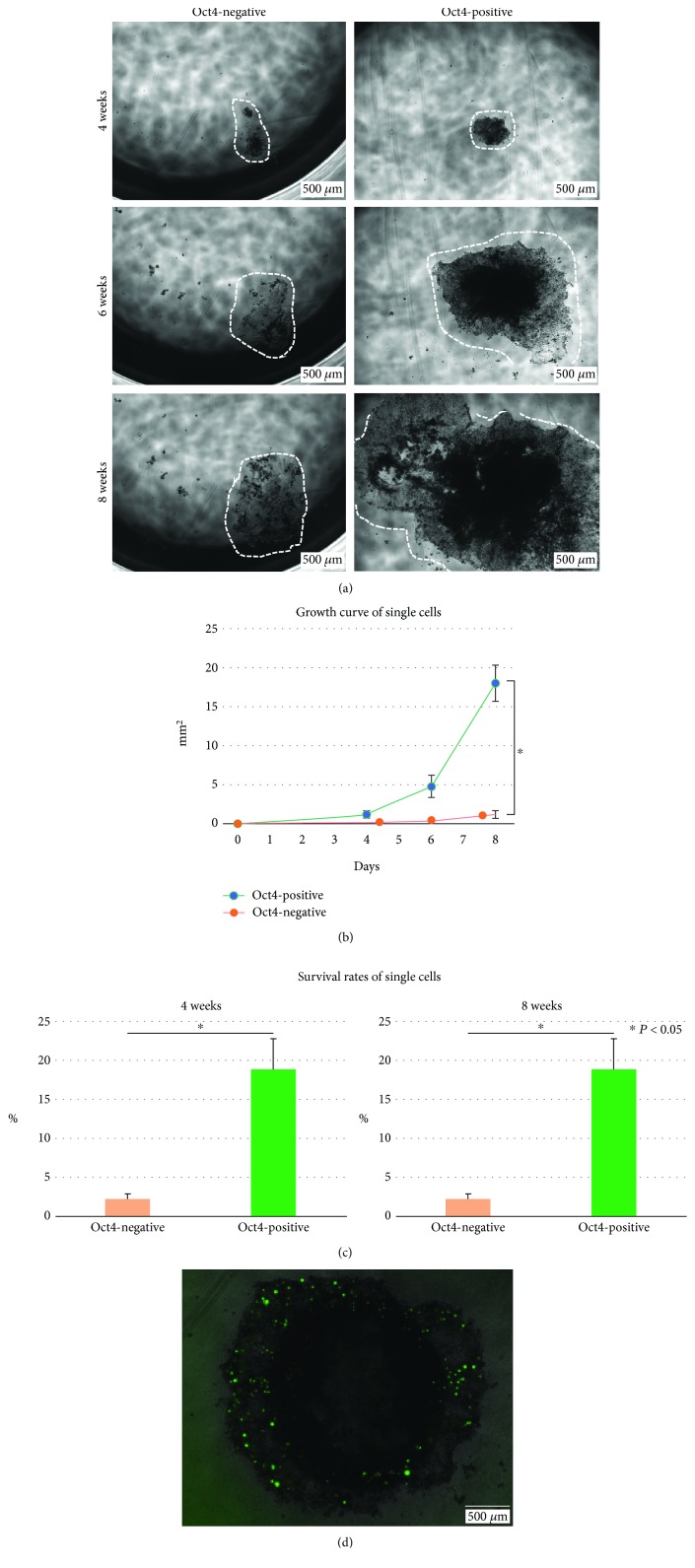
Growth of single sorted Oct4-positive and Oct4-negative cells. (a–c) Representative growth of Oct4-positive and Oct4-negative cells. (a) Growth images of Oct4-positive and Oct4-negative cells. Cells were indicated by white dotted lines. Oct4-positive cells proliferated well compared with Oct4-negative cells. (b) Growth curves of Oct4-positive and Oct4-negative cells. The colony sizes of Oct4-positive and Oct4-negative cells were measured. Oct4-positive cells proliferated well compared with Oct4-negative cells (*n* = 5, *P* < 0.05). (c) Survival rates of single sorted Oct4-positive and Oct4-negative cells. The survival rates of Oct4-positive cells were high compared with those of Oct4-negative cells four and eight weeks later of the sorting (*n* = 9, *P* < 0.005). (d) Representative image of colony from a single sorted Oct4-positive cell. Oct4-negative cells were produced from Oct4-positive cells. Scale bar, 500 *μ*m.

Oct4-EGFP-positive cells were enriched by sorting. Oct4-EGFP-positive cells reproduced the heterogenous population including Oct4-EGFP-negative cells (Figure 2(a)). The expression of *Oct4* mRNA was significantly higher in Oct4-EGFP-positive cells than in Oct4-EGFP-negative cells (Figure 2(b)). CD44 and CD133 have been reported previously as CRC stem cell markers [17, 18], and these markers were analyzed by flow cytometry. In the Oct4-EGFP-negative population, 76% of cells expressed CD44 and about 54% of cells expressed CD133 (Figure 2(c)). In the Oct4-high population, 98% of cells expressed CD44 and 54% of cells expressed CD133. All cells expressed CD44 and/or CD133 in the OCT4-high population. Moreover, gene set enrichment analysis (GSEA) showed that genes relating to WNT protein binding (*P* < 0.0001) and fibroblast growth factor (FGF) receptor binding (*P* < 0.0001) were enriched in Oct4-EGFP-high cells compared with Oct4-EGFP-negative cells (Figure 2(d), Supplementary Table S2). Next, Oct4-EGFP-positive and Oct4-EGFP-negative single cells were sorted into individual wells in the 96-well plate. Single sorted Oct4-EGFP-positive cells proliferated well compared with Oct4-EGFP-negative cells (Figures 3(a) and 3(b)). The number of wells with colonies was measured four and eight weeks later of the single-cell sorting. The survival rates of single cells were calculated as (number of wells with formed colony/number of sorted cells)×100 (%). Oct4-EGFP-positive single cells had significantly better survival than Oct4-EGFP-negative cells and kept long-time expansion (Figure 3(c)). Single sorted Oct4-high cells produced Oct4-EGFP-positive and Oct4-EGFP-negative cells (Figure 3(d)).

### 3.3. Liver Metastasis of Xenograft Model

**Figure 4:**
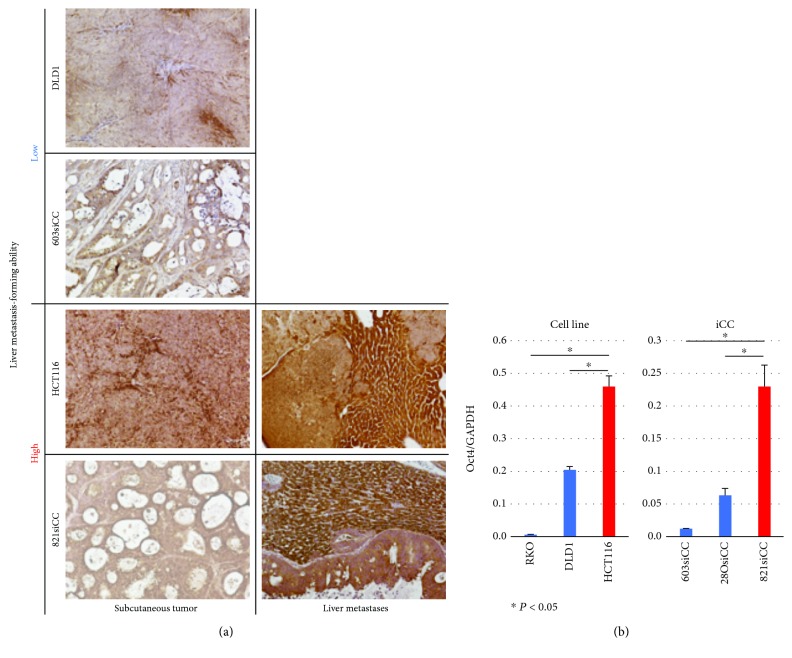
Oct4 protein and mRNA expression of cell lines and iCCs. (a) Representative staining of Oct4 in xenograft models: subcutaneous tumor and liver metastasis. Oct4 protein expression of subcutaneous tumor was high in HCT116 and 0821siCC, which had high liver metastasis-forming ability, compared with DLD1 and 603siCC. Oct4 protein expressions of liver metastasis formed by HCT116 and 821siCC were also high. DLD1 and 603siCC did not form liver metastasis. (b) *Oct4* mRNA expression was high in HCT116 and 821siCC compared with that in other cells (*n* = 3, *P* < 0.05). Scale bar, 100 *μ*m.

Three CRC cell lines (DLD1, HCT116, and RKO) and three CRC iCCs (603siCC, 28OsiCC, and 821siCC) were injected into the spleen to form liver metastasis (*n* = 4). The liver metastasis rate was 100% in 821siCC, 75% in HCT116, 25% in RKO, and 0% in DLD1, 603siCC, and 28OsiCC. 821siCC and HCT116 formed liver metastasis with high efficiency (≥75%). Oct4 protein expression and mRNA expression were examined using immunohistochemistry. RT-PCR, DLD1, 603siCC, and 28OsiCC did not form liver metastasis; therefore, Oct4 protein expression was compared using subcutaneous xenograft tumors. Oct4 protein expression of HCT116 and 821siCC was higher that of DLD1 and 603siCC (Figure 4(a)). Also, Oct4 protein expression in xenograft liver metastasis formed by HCT116 and 821siCC was high. *Oct4* mRNA expression of HCT116 and 821siCC was higher than that of other cells (RKO, DLD1, 603siCC, and 28OsiCC; Figure 4(b)).

## 4. Discussion

Metastasis occurs because of several combination factors, such as tumor location, tumor characteristics, and targeted organ characteristics [19–21]. Recent biological examination has shown that anti-EGFR monoclonal antibodies such as Cmab and Pmab are effective in wild-type RAS (KRAS/NRAS), and the drugs are selected according to the RAS mutation status without considering metastatic sites [3]. Although the present treatment for mCRC was the same in liver and/or lung metastasis patient targeted organ characteristics, targeted organ characteristics and their key factors remain poorly known. A better understanding of tumor characteristics will improve organ-specific treatment and prognosis for cancer patients. Overexpression of Oct4 and Nanog induces EMT and promotes metastasis of lung cancer [14], and knockdown of Oct4 suppresses EMT and blocks the metastatic ability in lung cancer and colorectal cancer [14, 15]. This is the first report to evaluate the relationship between Oct4 expression and liver metastasis of colorectal cancer (CRC) in clinical samples, the stemness of Oct4-expressed cells, and the liver metastasis-forming ability using primary cultured cells without genetic engineering.

We focused on liver metastasis of CRC and the Oct4 gene. *Oct4* expression was not significantly correlated with OS and DFS. However, high *Oct4* expression was significantly correlated with LMFS and it was an independent predictor of liver metastasis in CRC patients. The relationship between Oct4 expression and nonliver metastasis was also examined, but there was no significance. We revealed that Oct4 is a tumor characteristic that especially relates to liver metastasis in clinical CRC. Next, we examined the role of Oct4 with in vitro analysis focusing on the stemness. OCT4 can directly reprogram adult cells to induced pluripotent stem (iPS) cells, and it is also expressed in CRC CSCs [22, 23]. CSCs or “cancer stem-like cells” are thought to promote tumor cell invasion and metastasis [24] and to contribute to drug resistance [22, 25]. Primary cultured CRC cells are heterogenous compared with cell lines (Supplementary Figure S4), and the population of Oct4-EGFP-positive cells was examined in primary cultured cells. We established Oct4-EGFP primary cultured CRC cells and examined their characteristics. Single sorted Oct4-EGFP-positive cells proliferated and formed colonies more than Oct4-EGFP-negative cells, and Oct4-EGFP-positive cells produced Oct4-EGFP-positive and Oct4-EGFP-negative cells. These results show that Oct4-EGFP-positive cells have self-replication ability and self-propagation ability that were reported as the CSCs' characteristics [24]. Oct4-EGFP-positive cells more commonly expressed CD44/CD133 than Oct4-EGFP-negative cells, and all Oct4-high cells expressed CD44 [17]. GSEA showed that WNT protein binding and FGF receptor binding were enriched in Oct4-EGFP-high cells. The WNT signaling pathway plays an important role in CRC metastasis [26], and crosstalk of the FGF and WNT signaling pathways leads to a more malignant phenotype through several signaling cascades including EMT [27]. To summarize our findings, Oct4-EGFP-positive cells expressed more stem cell-associated markers compared with Oct4-EGFP-negative cells and had self-renewal and differentiation abilities like in CSCs. Moreover, primary cultured cells contain Oct4-expressed cells with self-renewal and differentiation abilities. Finally, we examined the capacity to form liver metastasis in vivo using CRC cell lines and primary cultured cells. Oct4 regulated epithelial-mesenchymal transition in CRC cell lines and its knockdown inhibited CRC cell migration and invasion [15]. We revealed that cells (HC116 and 821siCC) highly expressing Oct4 formed liver metastasis with high efficiency. This study has some limitations. In clinical analysis, the number of samples is too small to analyze nonliver metastasis. We did not examine metastatic potential to other sites such as lung metastasis, and more examination will need to reveal the role of Oct4 relating to organ-specific metastatic potential. However, we concluded that Oct4-high tumors might metastasize in a clinical context, so an additional therapeutic intervention for Oct4-high tumors and/or treatment to target Oct4 may reduce liver metastasis in CRC patients and improve their prognosis.

## 5. Conclusions

High *OCT4* expression was an independent predictor for liver metastasis in CRC patients. OCT4-positive primary cultured cells had self-renewal and differentiation abilities and actively formed liver metastasis.

## Figures and Tables

**Table 1:** Patient characteristics.

Factors	*N* = 173
Gender (male/female)	94/79
Age^∗^ (year)	65 (16–88)
CEA^∗^ (ng/mL)	3.6 (0.5–672.5)
Histological grade (Tub1/Tub2/other^∗∗^)	34/130/9
Tumor invasion (T2/T3/T4)	17/85/71
Lymph node metastasis (N0/N1/N2)	77/57/39
Lymphatic invasion (absent/present)	73/100
Vascular invasion (absent/present)	41/132
Stage (I/II/III/IV)	10/66/82/15

^∗^Continuous variable. ^∗∗^Other: poorly differentiated, mucinous adenocarcinoma, or squamous cell carcinoma. Tub1: well-differentiated adenocarcinoma; Tub2: moderately differentiated adenocarcinoma.

**Table 2:** Patient characteristics according to *Oct4* mRNA expression.

Factors	Low-expression group (*N* = 87)	High-expression group (*N* = 86)	*P* value
Age (<66/≥66)	46/41	42/44	0.595
Sex (male/female)	45/42	49/37	0.488
Preoperative CEA (≥5/<5)	39/46	33/51	0.386
Histological grade (other^∗^/Tub1–2)	4/83	4/82	0.987
Tumor invasion (T3–4/T2)	78/9	78/8	0.818
Lymph node metastasis (N1–2/N0)	45/42	51/35	0.316
Lymphatic invasion (present/absent)	52/35	48/38	0.598
Vascular invasion (present/absent)	61/26	71/15	0.054
TNM stage (1–2/3–4)	42/45	34/97	0.269

^∗^Other: poorly differentiated, mucinous adenocarcinoma, or squamous cell carcinoma. Tub1–2: well/moderately differentiated adenocarcinoma.

**Table 3:** Results of univariate and multivariate analyses of liver metastasis-free survival.

Factors	Univariate analysis			Multivariate analysis		
	HR	95% CI	*P* value	HR	95% CI	*P* value
Age (years) (<66/≥66)	1.008	0.484–2.112	0.983			
Sex (male/female)	1.696	0.805–3.798	0.167			
Preoperative CEA (≥5/<5)	1.277	0.526–2.732	0.526			
Histological grade (other^∗^/Tub1–2)	2.573	0.613–7.309	0.170			
Lymphatic invasion (present/absent)	2.085	0.959–5.012	0.064			
Vascular invasion (present/absent)	1.587	0.657–4.714	0.324			
**TNM stage (3-4/1-2)**	**8.232**	**2.896–34.536**	**<0.001**	**7.789**	**2.737–32.700**	**<0.001**
***Oct4* expression (high/low)**	**2.866**	**1.319–6.888**	**0.007**	**2.613**	**1.201–6.284**	**0.015**

^∗^Other: poorly differentiated, mucinous adenocarcinoma, or squamous cell carcinoma. Tub1: well-differentiated adenocarcinoma; Tub2: moderately differentiated adenocarcinoma.

## Data Availability

The data used to support the findings of this study are included within the article.

## Abbreviations

CRC: Colorectal cancer
OS: Overall survival
DFS: Disease-free survival
LMFS: Liver metastasis-free survival
mCRC: Metastatic CRC
Oct4 (POU5F1): POU domain, class 5, transcription factor 1
CSC: Cancer stem cells
EMT: Epithelial-mesenchymal transition
TNM classification: Tumor node metastasis classification
RT-PCR: Real-time reverse transcription
GSEA: Gene set enrichment analysis
iPS: Induced pluripotent stem.

## Supplementary Materials

Supplementary Materials Table S1: primer sequences corresponding to universal probe libraries. Table S2: pathways enriched in Oct4-EGFP-high cells. Figure S1: distribution of Oct4 mRMA expression levels in tumor samples. Figure S2: distribution of Oct4 mRMA expression levels stratified by liver metastasis status and TNM stage. Figure S3: survival curves for overall survival (OS) and disease-free survival (DFS) according to POU5F1 mRNA expression. Figure S4: flow cytometry analysis of CD24 and CD44 in cell lines and iCC Agilent microarray protocol.

## References

[1] Center for Cancer Control and Information Services NCC Japan recent cancer statistics, 2016. http://ganjoho.jp/reg_stat/statistics/stat/summary.html.

[2] Bray F., Ferlay J., Soerjomataram I., Siegel R. L., Torre L. A., Jemal A. (2018). Global cancer statistics 2018: GLOBOCAN estimates of incidence and mortality worldwide for 36 cancers in 185 countries. *CA: A Cancer Journal for Clinicians*.

[3] Van Cutsem E., Cervantes A., Nordlinger B., Arnold D., on behalf of the ESMO Guidelines Working Group (2014). Metastatic colorectal cancer: ESMO Clinical Practice Guidelines for diagnosis, treatment and follow-up. *Annals of Oncology*.

[4] Watanabe T., Muro K., Ajioka Y. (2018). Japanese Society for Cancer of the Colon and Rectum (JSCCR) guidelines 2016 for the treatment of colorectal cancer. *International Journal of Clinical Oncology*.

[5] Schmoll H. J., van Cutsem E., Stein A. (2012). ESMO consensus guidelines for management of patients with colon and rectal cancer. A personalized approach to clinical decision making. *Annals of Oncology*.

[6] Scholer H. R., Dressler G. R., Balling R., Rohdewohld H., Gruss P. (1990). Oct-4: a germline-specific transcription factor mapping to the mouse t-complex. *The EMBO Journal*.

[7] Loh Y. H., Wu Q., Chew J. L. (2006). The Oct4 and Nanog transcription network regulates pluripotency in mouse embryonic stem cells. *Nature Genetics*.

[8] Kim J. H., Jee M. K., Lee S. Y. (2009). Regulation of adipose tissue stromal cells behaviors by endogenic Oct4 expression control. *PLoS One*.

[9] Han S. M., Han S. H., Coh Y. R. (2014). Enhanced proliferation and differentiation of Oct4- and Sox2-overexpressing human adipose tissue mesenchymal stem cells. *Experimental & Molecular Medicine*.

[10] Miyoshi N., Fujino S., Ohue M. (2018). The POU5F1 gene expression in colorectal cancer: a novel prognostic marker. *Surgery Today*.

[11] Cai S., Geng S., Jin F., Liu J., Qu C., Chen B. (2016). POU5F1/Oct-4 expression in breast cancer tissue is significantly associated with non-sentinel lymph node metastasis. *BMC Cancer*.

[12] Guo X., Yu L., Zhang Z., Dai G., Gao T., Guo W. (2017). miR-335 negatively regulates osteosarcoma stem cell-like properties by targeting POU5F1. *Cancer Cell International*.

[13] Wang Y. D., Cai N., Wu X. L., Cao H. Z., Xie L. L., Zheng P. S. (2013). OCT4 promotes tumorigenesis and inhibits apoptosis of cervical cancer cells by miR-125b/BAK1 pathway. *Cell Death & Disease*.

[14] Chiou S. H., Wang M. L., Chou Y. T. (2010). Coexpression of Oct4 and Nanog enhances malignancy in lung adenocarcinoma by inducing cancer stem cell-like properties and epithelial-mesenchymal transdifferentiation. *Cancer Research*.

[15] Dai X., Ge J., Wang X., Qian X., Zhang C., Li X. (2012). OCT4 regulates epithelial-mesenchymal transition and its knockdown inhibits colorectal cancer cell migration and invasion. *Oncology Reports*.

[16] Sobin L. H. G. M., Wittekind C. (2010). *TNM Classification of Malignant Tumors*.

[17] Muraro M. G., Mele V., Däster S. (2012). CD133^+^, CD166^+^CD44^+^, and CD24^+^CD44^+^ phenotypes fail to reliably identify cell populations with cancer stem cell functional features in established human colorectal cancer cell lines. *Stem Cells Translational Medicine*.

[18] Du L., Rao G., Wang H. (2013). CD44-positive cancer stem cells expressing cellular prion protein contribute to metastatic capacity in colorectal cancer. *Cancer Research*.

[19] Guinney J., Dienstmann R., Wang X. (2015). The consensus molecular subtypes of colorectal cancer. *Nature Medicine*.

[20] Hoshino A., Costa-Silva B., Shen T. L. (2015). Tumour exosome integrins determine organotropic metastasis. *Nature*.

[21] Tejpar S., Stintzing S., Ciardiello F. (2017). Prognostic and predictive relevance of primary tumor location in patients with *RAS* wild-type metastatic colorectal cancer: retrospective analyses of the CRYSTAL and FIRE-3 trials. *JAMA Oncology*.

[22] Miyoshi N., Ishii H., Nagai K. (2010). Defined factors induce reprogramming of gastrointestinal cancer cells. *Proceedings of the National Academy of Sciences of the United States of America*.

[23] Miyoshi N., Ishii H., Nagano H. (2011). Reprogramming of mouse and human cells to pluripotency using mature microRNAs. *Cell Stem Cell*.

[24] Todaro M., Gaggianesi M., Catalano V. (2014). CD44v6 is a marker of constitutive and reprogrammed cancer stem cells driving colon cancer metastasis. *Cell Stem Cell*.

[25] Kong D., Li Y., Wang Z., Sarkar F. H. (2011). Cancer stem cells and epithelial-to-mesenchymal transition (EMT)-phenotypic cells: are they cousins or twins?. *Cancers*.

[26] Wang G., Fu Y., Yang X. (2016). Brg-1 targeting of novel miR550a-5p/RNF43/Wnt signaling axis regulates colorectal cancer metastasis. *Oncogene*.

[27] Katoh M., Katoh M. (2007). WNT signaling pathway and stem cell signaling network. *Clinical Cancer Research*.

