# Supplementary material for: Oct4 Gene Expression in Primary Colorectal Cancer Promotes Liver Metastasis
Source: Stem Cells Int. 2019 May 2;2019:7896524. doi: 10.1155/2019/7896524 (PMC6525814; doi:10.1155/2019/7896524)
Supplement: Supplementary Materials — Table S1: primer sequences corresponding to universal probe libraries. Table S2: pathways enriched in Oct4-EGFP-high cells. Figure S1: distribution of Oct4 mRMA expression levels in tumor samples. Figure S2: distribution of Oct4 mRMA expression levels stratified by liver metastasis status and TNM stage. Figure S3: survival curves for overall survival (OS) and disease-free survival (DFS) according to POU5F1 mRNA expression. Figure S4: flow cytometry analysis of CD24 and CD44 in cell lines and iCC Agilent microarray protocol. [file 7896524.f1.docx]

**Supplementary materials**

**Table S1**. **Primer sequences corresponding to universal probe libraries**

| Primer | Sequence 5′–3′ | UPL No. |
| --- | --- | --- |
| POU5F1-F | GCTTCAAGAACATGTGTAAGCTG | 69 |
| POU5F1-R | CACGAGGGTTTCTGCTTTG |  |
| GAPDH-F | AGCCACATCGCTCAGACAC | 60 |
| GAPDH-R | GCCCAATACGACCAAATCC |  |

**Table S2**. **Pathways enriched in Oct4-EGFP-high cells**

| Pathway | P value |
| --- | --- |
| Proximal distal pattern formation | <0.0001 |
| WNT protein binding | <0.0001 |
| Fibroblast growth factor receptor binding | <0.0001 |
| Positive regulation of IL8 production | <0.0001 |
| Homophilic cell adhesion via plasma membrane adhesion molecules | <0.0001 |
| Glutamate receptor activity | <0.0001 |

**Figure S1.** **Distribution of *Oct4* mRMA expression levels in tumor samples**


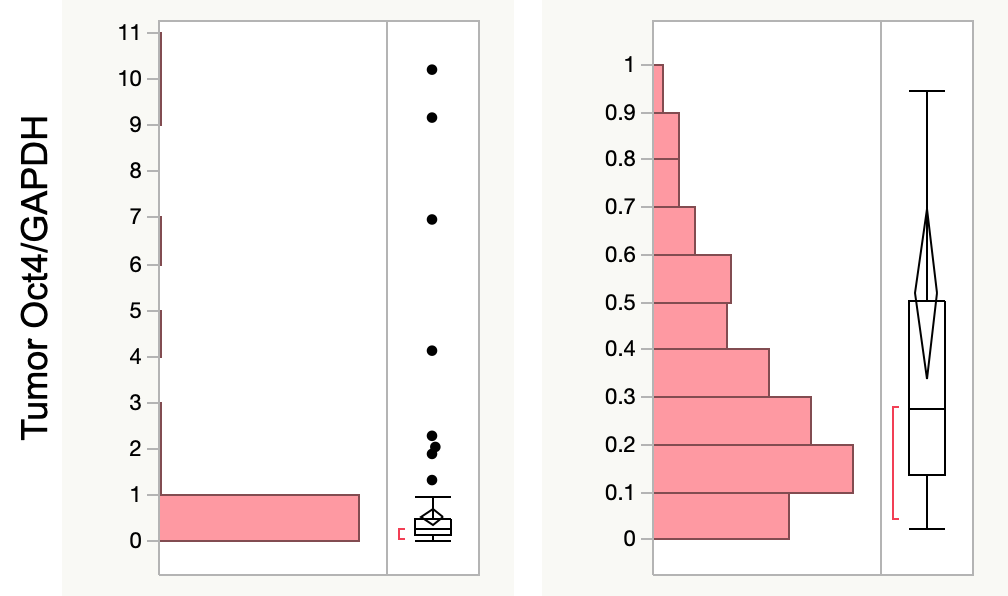


*Oct4* mRNA expression levels were calculated as *Oct4*/*GAPDH* expression for each sample. The median value of the *Oct4/GAPDH* mRNA expression level was 0.273 (range, 0.021-10.187).

**Figure S2.** **Distribution of *Oct4* mRMA expression levels stratified by liver metastasis status and TNM stage.**


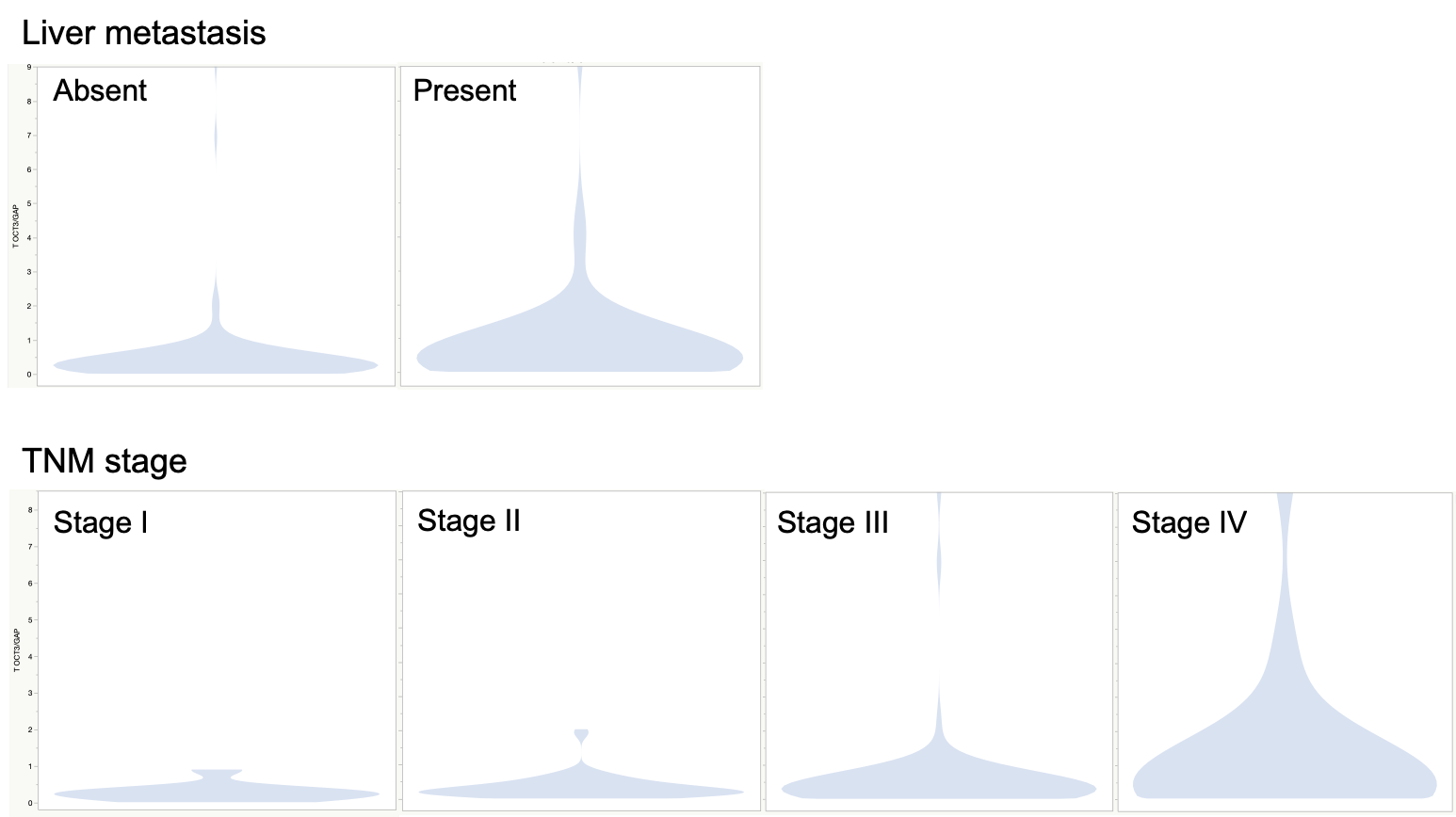


The *Oct4/GAPDH* mRNA expression was high in patients with liver metastasis. The Oct4/GAPDH ratio of oct4 was elevated as the stage increased.

**Figure S3.** **Survival curves for overall survival** (**OS) and disease-free survival (DFS) according to *POU5F1* mRNA expression**

The 5-year OS rate was 83% (n=87) in the low-expression group and 77% (n=86) in the high-expression group (P=0.464). The 5-year DFS rate was 77% (n=87) in the low-expression group and 67% (n=86) in the high-expression group (P=0.185).

**Figure S4.** **Flow cytometry analysis of CD24 and CD44 in cell lines and iCCs**


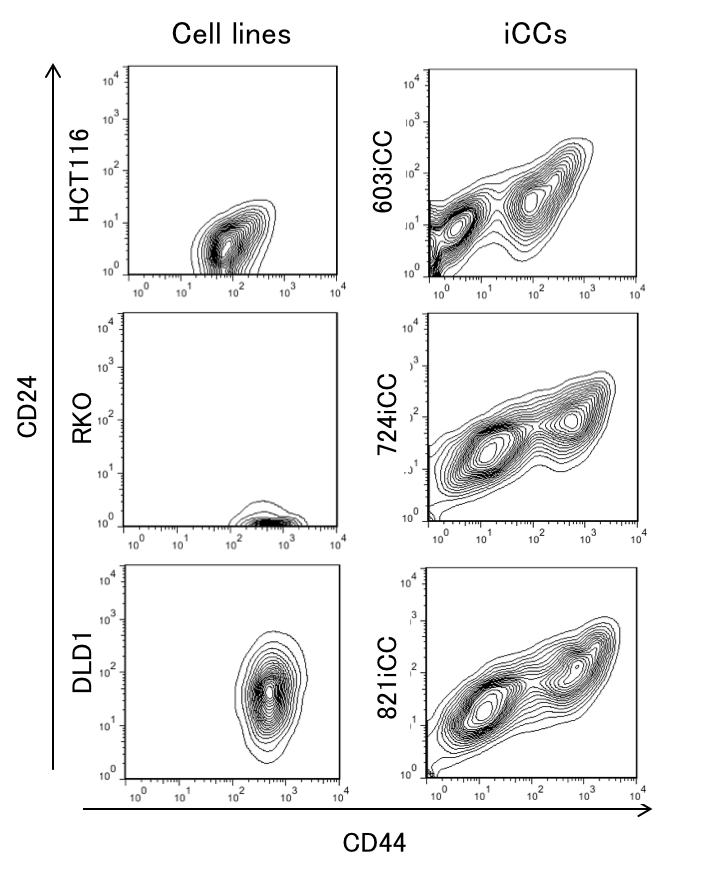


iCCs had CD24 low/high and CD44 low/high population. Cell lines show the homogenous population compared with iCCs.

***Agilent microarray protocol***

Cyanine-3 (Cy3)-labelled cRNA was prepared from 0.1 µg of total RNA using a Low Input Quick Amp Labeling Kit (Agilent, Santa Clara, CA, USA) according to the manufacturer’s instructions, followed by purification using an RNeasy column (Qiagen, Valencia, CA, USA). Dye incorporation and cRNA yields were determined using a NanoDrop ND-2000 Spectrophotometer (Thermo Fisher Scientific, Waltham, MA, USA). Cy3-labelled cRNA (0.6 µg) was fragmented at 60°C for 30 min in 25 µl of 1× Agilent fragmentation buffer and 2× Agilent blocking agent according to the manufacturer’s instructions. Next, 2× Agilent hybridization buffer was added to the fragmentation mixture and hybridized with a SurePrint G3 Human GE 8×60K Microarray v2 (Agilent) for 17 h at 65°C in a rotating Agilent hybridization oven. After hybridization, the microarrays were washed with GE Wash Buffer 1 (Agilent) for 1 min at room temperature and for 1 min at 37°C with GE Wash Buffer 2 (Agilent). Immediately after washing, the slides were scanned using and Agilent SureScan Microarray Scanner (G2600D) set for one-color scanning of 8 × 60 K array slides (scan area = 61 × 21.6 mm, resolution = 3 µm, dye channel = green, and photomultiplier tube set to 100%). The scanned images were analysed using Feature Extraction Software 11.5.1.1 (Agilent) and the default parameters to obtain background subtracted and spatially detrended processed signal intensities.
